# Yoga for older adults with multimorbidity (the Gentle Years Yoga Trial): study protocol for a randomised controlled trial

**DOI:** 10.1186/s13063-021-05217-5

**Published:** 2021-04-12

**Authors:** Garry A. Tew, Laura Bissell, Belen Corbacho, Caroline Fairhurst, Jenny Howsam, Jess Hugill-Jones, Camila Maturana, Shirley-Anne S. Paul, Tim Rapley, Jenny Roche, Fi Rose, David J. Torgerson, Lesley Ward, Laura Wiley, David Yates, Catherine Hewitt

**Affiliations:** 1 Department of Sport, Exercise and Rehabilitation, Northumbria University, Newcastle-upon-Tyne, UK; 2 York Trials Unit, Department of Health Sciences, University of York, York, UK; 3 British Wheel of Yoga Qualifications (BWYQ), Sleaford, Lincs, UK; 4 Department of Social Work, Education and Community Wellbeing, Northumbria University, Newcastle-upon-Tyne, UK; 5 Department of Anaesthesia, York Hospitals NHS Foundation Trust, York, UK

**Keywords:** Aged, Multimorbidity, Mind-body therapies, Health-related quality of life, Randomised controlled trial

## Abstract

**Background:**

Multimorbidity is common in older adults and associated with high levels of illness burden and healthcare expenditure. The evidence base for how to manage older adults with multimorbidity is weak. Yoga might be a useful intervention because it has the potential to improve health-related quality of life, physical functioning, and several medical conditions. The British Wheel of Yoga’s Gentle Years Yoga© (GYY) programme was developed specifically for older adults, including those with chronic medical conditions. Data from a pilot trial suggested feasibility of using GYY in this population, but its effectiveness and cost-effectiveness remain uncertain.

**Methods:**

This is a multi-site, individually randomised, superiority trial with an embedded process evaluation and an economic analysis of cost-effectiveness. The trial will compare an experimental strategy of offering a 12-week GYY programme against a control strategy of no offer in community-dwelling adults aged 65 or over who have multimorbidity, defined as having two or more chronic conditions from a predefined list. The primary outcome is health-related quality of life measured using the EQ-5D-5L, the primary endpoint being the overall difference over 12 months. Both groups will continue to be able to access their usual care from primary, secondary, community, and social services. Participants, care providers, and yoga teachers will not be blinded to the allocated intervention. Outcome measures are primarily self-reported. The analysis will follow intention-to-treat principles.

**Discussion:**

This pragmatic randomised controlled trial will demonstrate if the GYY programme is an effective, cost-effective, and viable addition to the management of older adults with multimorbidity.

**Trial registration:**

ISRCTN ISRCTN13567538. Registered on 18 March 2019

## Administrative information

**Table:** 

Title {1}	Yoga for older adults with multimorbidity (the Gentle Years Yoga Trial): study protocol for a randomised controlled trial
Trial registration {2a and 2b}.	ISRCTN13567538. Prospectively registered on 18th March 2019.
Protocol version {3}	Version 1.5; 09/07/2020
Funding {4}	National Institute for Health Research, Health Technology Assessment programme, project number 17/94/36
Author details {5a}	^1^Department of Sport, Exercise and Rehabilitation, Northumbria University, Newcastle-upon-Tyne, UK ^2^York Trials Unit, Department of Health Sciences, University of York, York, UK ^3^British Wheel of Yoga Qualifications (BWYQ), Sleaford, Lincs, UK ^4^Department of Social Work, Education and Community Wellbeing, Northumbria University, Newcastle-upon-Tyne, UK ^5^Department of Anaesthesia, York Hospitals NHS Foundation Trust, York, UK
Name and contact information for the trial sponsor {5b}	Northumbria University, ethicssupport@northumbria.ac.uk
Role of sponsor {5c}	The sponsor played no part in study design; and will play no part in the collection, management, analysis, and interpretation of data; writing of the report; and the decision to submit the report for publication.

## Introduction

### Background and rationale {6a}

Multimorbidity, defined as when a person has two or more long-term medical conditions, is one of the biggest challenges facing health systems internationally as multiple disease care becomes commonplace in an ageing society [1, 2]. It is highly prevalent in older adults, with one study showing 65% of adults aged 65–84 years to be multimorbid [3]. Multimorbidity leads to poorer health outcomes: it is associated with reduced life expectancy, quality of life, and physical and mental well-being [4, 5]. Multimorbid individuals also consume a disproportionally large share of healthcare resources [6, 7].

The evidence base for enhancing the care of patients with multimorbidity is limited [8, 9]. A 2016 Cochrane review found only 18 randomised controlled trials that had evaluated interventions for improving outcomes in patients with multimorbidity in primary care and community settings [9]. In 12 studies, the interventions were primarily organisational, e.g. case management or addition of a pharmacist to the clinical care team. In the other six studies, the interventions were primarily patient-oriented, e.g. self-management support groups. Across all studies, there was moderate certainty evidence of little or no difference in clinical outcomes and high certainty evidence of mental health outcomes improving (in the intervention versus control). There was moderate certainty evidence of a small improvement in patient-reported outcomes, and two trials that specifically targeted patients’ functional difficulties showed positive effects on functional outcomes [10, 11]. There was limited data on costs. This review highlighted the need for further research to determine the clinical and cost-effectiveness of interventions that are ideally simple, generalisable, and which can address several medical conditions simultaneously. Yoga is a candidate intervention.

Yoga originated thousands of years ago in India as an integrated mind-body practice based on ancient Vedic philosophy. During the twentieth century, yoga became increasingly recognised outside India, and over the past decades, it has continued to grow in popularity worldwide as a system for promoting health and well-being. Whilst modern yoga often focuses on physical poses and is sometimes thought of as a type of exercise, the practice usually incorporates one or more of the mental or spiritual elements that are traditionally part of yoga, such as relaxation, concentration, or meditation. There are currently many different styles or schools of yoga, each with a variable emphasis and approach to practice. Research evidence suggests that some of these yoga practices may help to prevent and treat various physical and mental illnesses and improve the overall quality of life [12].

In November 2017, the Cochrane Library published a special collection of 14 systematic reviews that focused on the effectiveness of yoga for improving physical or mental symptoms and quality of life in a range of health conditions, including musculoskeletal, pulmonary, cancer, cardiovascular, neurological, and mental health. A summary of four of these reviews is as follows:

- Yoga for chronic non-specific low back pain [13]: For yoga compared to non-exercise controls (9 trials; 810 participants), there was moderate certainty evidence that yoga produced small-to-moderate improvements in back-related function (standardised mean difference [SMD] −0.44, 95% confidence interval [CI] −0.66 to − 0.22) and pain (mean difference [MD] −7.81, 95% CI − 13.37 to − 2.25) at 6 months. The authors recommended additional high-quality research to improve confidence in estimates of effect and to evaluate long-term outcomes.
- Yoga for asthma [14]: There was some evidence that yoga may improve quality of life (MD in Asthma Quality of Life Questionnaire score per item 0.57 units on a 7-point scale, 95% CI 0.37 to 0.77; 5 studies; *n* = 375) and symptoms (SMD 0.37, 95% CI 0.09 to 0.65; 3 studies; *n* = 243) and reduce medication usage (risk ratio 5.35, 95% CI 1.29 to 22.11; 2 studies) in people with asthma. The authors concluded that large high-quality trials are needed to confirm the effects of yoga on asthma.
- Yoga for improving health-related quality of life, mental health, and cancer-related symptoms in women diagnosed with breast cancer [15]: Seventeen studies that compared yoga versus no therapy provided moderate-quality evidence showing that yoga improved health-related quality of life (SMD 0.22, 95% CI 0.04 to 0.40; 10 studies, *n* = 675), reduced fatigue (SMD −0.48, 95% CI − 0.75 to − 0.20; 11 studies, *n* = 883), and reduced sleep disturbances in the short term (SMD -0.25, 95% CI − 0.40 to − 0.09; six studies, *n* = 657). No serious adverse events were reported. Additional research is needed to assess the medium- and longer-term effects.
- Yoga for primary prevention of cardiovascular disease [16]: Yoga was found to produce reductions in diastolic blood pressure (MD − 2.90 mmHg), triglycerides (MD − 0.27 mmol/L), and high-density lipoprotein cholesterol (MD 0.08 mmol/L). There was no clear evidence of a difference between groups for low-density lipoprotein cholesterol, although there was moderate statistical heterogeneity. Adverse events, occurrence of type 2 diabetes, and costs were not reported in any of the studies. No study reported cardiovascular mortality, all-cause mortality, or non-fatal events, and most studies were small- and short-term.

Elsewhere, studies have sought to determine the effects of yoga in older populations. For example, a 2012 systematic review of 16 studies (*n* = 649) [17] and a more recent trial of 118 participants [18] demonstrated that yoga may provide greater improvements in physical functioning and self-reported health status than conventional physical activity interventions in older adults. More recently, a systematic review of six trials (*n* = 307) of relatively high methodological quality reported that yoga interventions had a small beneficial effect on balance (SMD 0.40, 95% CI 0.15 to 0.65, 6 trials) and a medium effect on physical mobility (SMD 0.50, 95% CI 0.06 to 0.95, 3 trials) in people aged 60 and over [19].

In summary, these data offer support for the beneficial effects of yoga in older adults and for several long-term medical conditions. However, many of the previous studies had limitations, including small sample sizes, a single yoga teacher delivering the programme, and short-term follow-up. Robust economic evaluations of yoga are also limited, although a recent systematic review concluded that ‘medical’ yoga is likely to be a cost-effective option for low back pain [20]. Very little research has specifically focused on older adults with multimorbidity.

In 2009, the Gentle Years Yoga (GYY) programme was developed by the Yorkshire Yoga & Therapy Centre to cater specifically for the needs of older adults, including those with long-term medical conditions such as osteoarthritis, hypertension, and cognitive impairment. As part of the pilot research study conducted at Yorkshire Yoga in 2016, a standardised GYY teacher training programme was manualised with the creation of a quality-assured teacher training course which became the British Wheel of Yoga (BWY) Gentle Years Yoga© (GYY) programme that is being delivered by the BWY. BWY is the national governing body of yoga in Great Britain with a nationwide network of > 5000 qualified yoga teachers. GYY is based on standard Hatha Yoga, incorporating traditional physical poses and transitions as well as breathing, concentration, and relaxation activities. Adaptations to challenging Hatha Yoga poses have been made so that older adults can safely participate whilst still obtaining the fitness, health, and well-being benefits of yoga. Each programme involves one group-based session per week for 12 weeks (each session including a 75-min chair-based yoga class and after-class social time) and promotion of regular self-managed yoga practice at home.

In a pilot trial of the GYY programme [21], 82 potential participants (community-dwelling inactive older adults) expressed an interest within a 2-month recruitment period, of which 52 (mean age 75 years) were recruited and randomised. Participants had up to six long-term medical conditions, the most common of which were osteoarthritis, hypertension, and depression. Trial yoga courses were delivered across four community venues by four yoga teachers. Two thirds (67%) of participants had an acceptable attendance of ≥80%. Feasibility was demonstrated, with potential for a positive clinically important effect on health status (EQ-5D-5L utility score) at 3 months after randomisation (MD 0.12, 95% CI 0.03 to 0.21). We are now conducting a larger trial over a wider geographical area to ascertain the clinical and cost-effectiveness of the intervention in older adults with multimorbidity.

### Objectives {7}

The primary objective is to establish if the offer of a 12-week GYY programme in addition to continued access to usual care is more effective compared with usual care alone in improving health-related quality of life (EQ-5D-5L utility index score) over 12 months in adults aged 65 years or over with multimorbidity.

Secondary objectives include the following:

- To explore the effect of the GYY programme on health-related quality of life, depression, anxiety, and loneliness at 3, 6, and 12 months after randomisation
- To explore the effect of the GYY programme on the incidence of falls over 12 months after randomisation
- To explore the safety of the GYY programme relative to control in terms of the occurrence of adverse events over 12 months after randomisation
- To assess if the GYY programme is cost-effective, measured using differences in cost of health resource use between the intervention and control groups and the incremental cost-effectiveness ratios using quality-adjusted life years (QALYs) derived from the EQ-5D-5L measured at 3, 6, and 12 months after randomisation
- To undertake a qualitative process evaluation to describe the experience of the intervention, explain the determinants of delivery (including treatment fidelity), and identify the optimal implementation strategies for embedding and normalising the GYY programme in preparation for a wider roll-out

### Trial design {8}

The present study is a multi-site, parallel-group, superiority, individually randomised controlled trial comparing an experimental strategy of offering a 12-week GYY programme against a control strategy of no offer in community-dwelling adults aged 65 or over who have multimorbidity. Both trial arms will continue to be able to access their usual care from primary, secondary, community, and social services.

The research design also includes a qualitative process evaluation, an economic analysis of cost-effectiveness, and four methodological sub-studies that will address the following questions:

- What is the concurrent validity of the 29-item Patient-Reported Outcomes Measurement Information System® (PROMIS-29) with the EQ-5D-5L?
- Does including £5 and/or a pen in the recruitment pack enhance recruitment?
- Does sending a pen with a follow-up questionnaire enhance return rates?
- Does offering a free yoga session to control participants after the 12-month follow-up assessment enhance retention and reduce contamination?

The procedures for the methodological sub-studies have been posted on a repository hosted by the Northern Ireland Network for Trials Methodology Research ([22]; see studies 92–95).

## Methods: participants, interventions, and outcomes

### Study setting {9}

We are recruiting patients from primary care and the community in England, Wales, and Scotland. The yoga courses will be delivered either face-to-face in a non-medical community-based facility (e.g. yoga studio, community hall, leisure centre), or online via video conferencing during periods of social distancing restrictions resulting from the COVID-19 pandemic.

### Eligibility criteria {10}

Patients will be eligible to join the study if they are aged 65 years or older (both male and female), community-dwelling (including sheltered housing living with support), and have two or more of the following chronic conditions:

- Arthritis, including osteoarthritis, rheumatoid arthritis, and history of shoulder, hip, or knee arthroplasty for arthritis
- Asthma or chronic obstructive pulmonary disease
- Atrial fibrillation
- Bowel problems, including irritable bowel syndrome, diverticulitis, and inflammatory bowel disease
- Cancer, diagnosed within the last 5 years
- Cardiovascular disease, including coronary heart disease, hypertension, heart failure, and peripheral arterial disease
- Chronic kidney disease
- Dementia (only if patients have the capacity to provide written informed consent)
- Depression or anxiety
- Diabetes
- Epilepsy
- Fibromyalgia
- Multiple sclerosis
- Osteoporosis or osteopenia
- Parkinson’s disease
- Sensory conditions, including hearing loss, macular degeneration, cataracts, and glaucoma
- Stroke, within the last 5 years

Patients will not be eligible for the study if they meet one or more of the following exclusion criteria:

- Inability to attend one of the yoga courses on offer*
- Attended yoga classes twice a month or more in the previous 6 months
- Contraindications to yoga participation (as identified by the patient’s general practitioner [GP])
- Severe mental health problem: schizophrenia, bipolar affective disorder, or other psychotic illnesses
- Learning disability
- Unable to read or speak English
- Unable to provide consent
- Unable to complete and return a valid baseline questionnaire
- No more than one patient per household
- Currently enrolled in another research study for which concurrent participation is deemed inappropriate by their GP or a clinician co-investigator

*Participants need to be available to attend at least 9 of the 12 classes on offer. In relation to online classes, factors that might make someone unable to participate include no internet access, unfamiliarity with or inability to use the internet, no suitable device for accessing the online classes (e.g. tablet-sized screen or larger; device with camera and microphone), insufficient space at home, and no sturdy chair for use in the classes. All eligibility criteria are assessed by reviewing responses to specific questions posed in a screening questionnaire that is self-completed by potential participants either in written format or over the telephone with a researcher.

#### Eligibility criteria for primary care general practices

To be eligible for inclusion as a recruitment centre, the practices need to be located close to (e.g. ideally within 5 miles of) the yoga class venue (for face-to-face classes) and to use EMIS Web or SystmOne as their computer system. The selection of general practices is also informed by list size, local transport routes, teacher recommendations, and staff availability for conducting recruitment activities.

#### Eligibility criteria for yoga teachers

To be eligible for inclusion, yoga teachers need to have completed the GYY qualification and have valid BWY membership and insurance. For online courses, teachers also need to be proficient in remote teaching. The selection of yoga teachers is informed by observations of them leading non-trial yoga classes, as conducted by the trial’s yoga consultants (LB and JH).

### Who will take informed consent? {26a}

Potentially eligible patients are posted a recruitment pack, which includes an invitation to participate and an information sheet that provides a balanced written account of the purpose and design of the trial. The information sheet also includes details of who to contact to ask any questions and how they can access an audio recording of the information sheet on the trial website. A simple diagram of the trial design is also provided with the information sheet to facilitate participants’ understanding of the randomisation process and what each group receives.

Potential participants provide consent either by completing a hard copy of the consent form and returning it to the trial team via post or by submitting an electronic consent form. Participants indicate on the consent form if they would like to be contacted to discuss possible interviews for the process evaluation. If the participant indicates ‘yes’, the process evaluation researcher provides the participant with an information sheet regarding the process evaluation interviews and obtains additional written consent from those who agree to take part. Individuals who decline to participate in the trial may indicate a willingness to take part in the process evaluation interviews as a ‘trial decliner’.

### Additional consent provisions for collection and use of participant data and biological specimens {26b}

The consent form asks if participants consent to the information that is collected about them being used to support other research in the future, and to it potentially being shared anonymously with other researchers.

## Interventions

### Explanation for the choice of comparators {6b}

The comparator is usual care alone. Usual care is defined as ‘the wide range of care that is provided in a community whether it is adequate or not, without a normative judgement’ [23]. Both trial arms will continue to be able to access their usual care from primary care, secondary care, community, and social services. The comparator (i.e. usual care alone) is relevant since it acknowledges the main aim of this pragmatic trial: to determine the effectiveness and cost-effectiveness of offering the GYY programme in addition to usual care.

### Intervention description {11a}

**Fig. 1:**
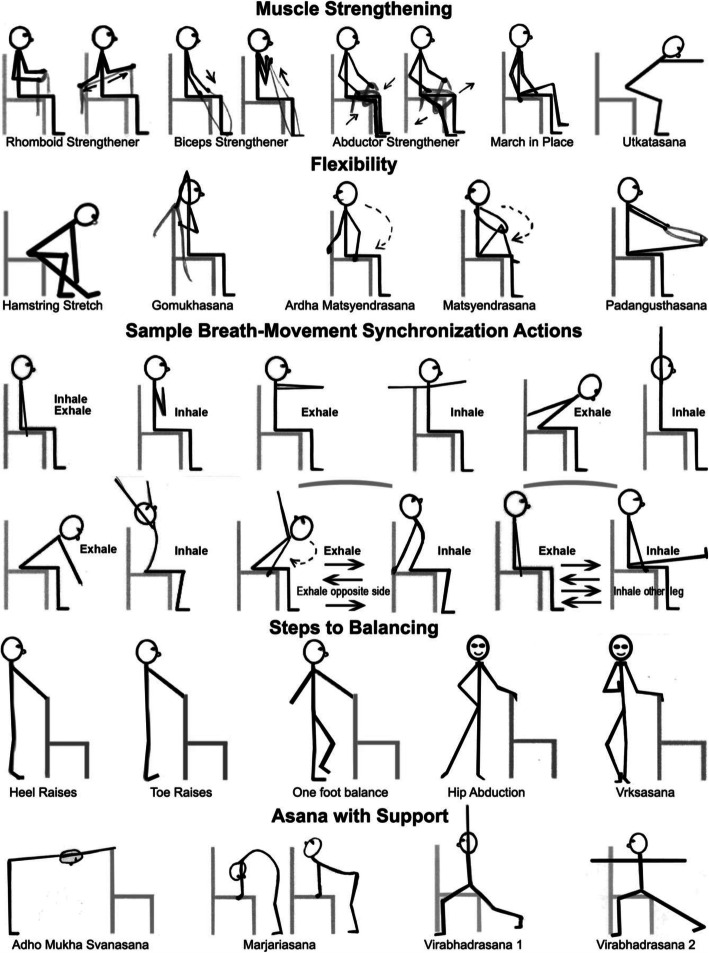
Sample of chair-based postures that are commonly used in GYY classes. Reproduced with permission from Tew et al. [21]

GYY is a yoga programme for older adults, including those with long-term medical conditions. It is based on standard Hatha Yoga and incorporates traditional physical postures and transitions as well as breathing, concentration, and relaxation activities. Each class also includes additional time immediately following the class for participants to stay on and socialise. The main aims of the programme are to strengthen the muscles, increase flexibility, balance, and mobility, and improve mental and social well-being. Chairs are used for seated exercise and can be used to provide support when standing, although the whole session can be carried out on a chair. Figure 1 shows examples of seated poses that are commonly used. The yoga practices are modified, where necessary, to allow individuals with varying medical conditions and functional abilities to participate safely. Props are also sometimes used to modify some of the postures and concentration activities. The physical challenge of each posture will be progressed throughout the course as participants become more able and confident. The following bullet points summarise how the GYY classes differ from standard Hatha Yoga classes:

- For the most part, participants are seated on chairs, and when standing, they use the chair or other aids for support.
- The classes do not use supine, semi-supine, or prone postures; instead, the key elements of traditional supine/prone postures are integrated into seated or standing postures.
- The classes hold static postures for a shorter length of time, especially those that could cause more pronounced acute increases in blood pressure.
- The physical set-up of classes has been adapted to suit people with sensory impairment, specifically participants being relatively close to the teacher, lighting levels being higher, the colour of equipment being in contrast to that of the walls and floor, and no music played during verbal instructions.
- The pace and overall structure of the class allow greater time for recovery from the more intense activities (e.g. by having a simple breathing practice follow a more challenging physical posture)
- If there are individuals with cognitive impairment in the group, the teacher will use short, single-subject phrases and pace the instructions to allow time for processing each element of the instructions
- Longer warm-up and overall slower pace, making it safer for older adults and at a level where they can work without feeling ‘left behind’ or ‘too old for yoga’, or having their self-confidence eroded
- Breathing practices avoid retention, as this is contraindicated for individuals with hypertension.
- Mobilisation, postures, and concentration activities are incorporated that specifically focus on balance and coordination.

Participants will be invited to take part in 12, free of charge, 75-min group-based GYY classes. Each class is immediately followed by an optional 15–30-min period of socialising. All courses will commence within 3 weeks after randomisation. There will usually be one class per week for 12 consecutive weeks; however, there is an allowance for a gap during public holidays or if the teacher needs to be away for a week or two. Before the first class, participants are required to complete and submit a BWY Health Questionnaire. The questionnaire is needed for BWY teaching insurance purposes and so that the teachers are aware of each participant’s medical conditions and activity status. After the course starts, each class will include the following: (i) ‘housekeeping’ activities, such as completing the class register and discussing any home practice or health issues (5 min); (ii) introduction to the theme and practices of the class, basic breathing, and focusing activities (5 min); (iii) extended warm-up/mobilisation, preparatory postures (30–35 min); (iv) focused postures and restorative activities (10–15 min); (v) breathing exercises (5–10 min); and (vi) relaxation and concentration activities (5–10 min), followed by after-class social time (15–30 min).

In addition to lessons modified for the individuals within the class, the participants are instructed in chair-based yoga activities selected for safe self-practice at home. Participants are encouraged to practice at home each day using a home practice sheet. As the class-based activities become more challenging, students are given new home practice sheets to allow the progression of their home yoga routine. Each yoga participant will receive four home practice sheets in total, covering weeks 1–3, 4–6, 7–9, and 10–12. Each sheet includes at least five practices, and each home practice session is expected to last 10–20 min. Towards the end of the course (i.e. class 11 or 12), the yoga teachers will provide participants with general verbal advice about continuing yoga practice (including home practice) and a written or electronic one-page handout sign-posting participants to suitable yoga classes (i.e. GYY or similar) in their local community or online, which they may want to attend on a self-pay basis.

The delivery mode of the 12-week intervention will be either face-to-face or online. Online classes address the need for social distancing during the COVID-19 pandemic. The trial classes will comprise intervention participants only, with each 12-week course having 12 participants allocated if delivered online and up to 15 participants if delivered face-to-face. Face-to-face classes will be conducted in non-medical community-based facilities (e.g. yoga studio, community hall, leisure centre) following checks of venue suitability by the trial team. Accessibility factors to consider include close proximity to public transport links, parking facilities, and disability access. Online classes will be conducted via Zoom, an online livestream platform freely accessible to participants using a computer or tablet. Participants will be advised to activate their cameras so that the teacher is able to view them and provide feedback on their technique. Prior to online classes commencing, the yoga teacher will conduct one-to-one Zoom meetings with their students to optimise the set-up of their equipment and environment and to discuss any health issues or course queries. Participants will also be able to ask questions throughout the course.

#### Yoga teacher training

Approximately 24 yoga courses will be delivered within the trial by approximately 12 yoga teachers (i.e. each teacher delivering two separate courses). The teachers will all have the GYY qualification, appropriate insurance, and experience of working with older adults. They will also receive specific training in the trial background and procedures.

The GYY programme is copyrighted by the BWY and since 2017 has been providing training in GYY to qualified yoga teachers. Training for the GYY qualification takes place over approximately 12 months and covers the National Occupational Standards for understanding the principles of adapting physical activity for older adults and the planning, adaptation, and delivery of sessions to meet the requirements of participants with specific needs. This includes information on ageing, exercise barriers and motivators, ethical and legal responsibilities, the physiology of ageing and common chronic conditions, and how to modify yoga for different health states. After distance learning modules and face-to-face instruction, the teachers demonstrate their understanding through worksheets, multiple-choice questions, two case studies, designing a GYY programme, and being observed and assessed on their teaching of GYY sessions on two occasions.

To minimise inter-teacher variation and enhance the fidelity of intervention delivery, the yoga teachers will receive standardisation training from the trial team via a 1-day interactive workshop. The training will include clinical reasoning for the GYY programme, clarification of the standardised class content and structure, and practical delivery tips including intervention progression and provision of home practice sheets. It will also stress the importance of only allowing people in the trial’s intervention group to access the classes and other trial processes such as adverse event reporting and class attendance monitoring. To supplement this training day, teachers will also receive a standardised research training manual. The yoga teachers will have continued access to the intervention supervisors who developed the GYY teacher training course during their delivery of the 12-week intervention. The intervention supervisors will be responsible for supporting intervention delivery and sharing of best practice. Additional training and support will be available from the wider trial team as required, and this will be documented.

### Criteria for discontinuing or modifying allocated interventions {11b}

There are no specific criteria for discontinuing or modifying allocated interventions. Participants may choose to stop doing the yoga programme themselves for any reason or may be advised by their medical practitioner to discontinue practice due to ill health.

### Strategies to improve adherence to interventions {11c}

To optimise and encourage attendance, the teachers will be asked to contact participants who miss two consecutive classes without prior notification.

### Relevant concomitant care permitted or prohibited during the trial {11d}

Usual care for participants continues throughout the trial. There is nothing prohibited.

### Provisions for post-trial care {30}

Towards the end of their 12-session course, yoga participants will receive details of GYY or other suitable yoga classes that they could join on a self-pay basis. The control participants will receive the same information after completing the final follow-up questionnaire (12 months). There are no special compensation arrangements for those who suffer non-negligent harm from trial participation.

### Outcomes {12}

#### Primary outcome measure—EuroQoL 5 Dimensions (5L) Score (EQ-5D-5L)

The primary outcome will be health-related quality of life (HRQoL) as measured by the EQ-5D-5L utility score [24]. This will be assessed according to current EuroQol guidance [25] at baseline and 3, 6, and 12 months after randomisation. The primary endpoint will be the overall difference over the 12 months. There is a valuation set for the EQ-5D-5L available for England [26]; however, this is currently under revision. Meanwhile, the UK’s National Institute for Health and Care Excellence (NICE) recommends that utility values should be calculated using the crosswalk developed by van Hout et al. [27]. Utility scores will be calculated following the NICE guidance at the time of the analysis.

The EQ-5D™ is a widely used self-reported generic measure of HRQoL which comprises two parts: the classification of 5 dimensions of health (mobility, self-care, usual activities, pain/discomfort, and anxiety/depression) and a visual analogue scale (VAS), which records participants’ overall evaluation of their health on a scale from 100 (best imaginable health) to 0 (worst imaginable health). The EQ-5D has been validated in many different patient populations including diabetes, cardiovascular problems, chronic obstructive pulmonary disease, cancer, chronic pain, and rheumatoid arthritis. There are currently two versions of the instrument that can be used for adults: the original EQ-5D-3L with five dimensions of health and 3 levels of problems, and the more recent EQ-5D-5L that has the same five dimensions of health and 5 levels of problems (1 = no problems, 2 = slight problems, 3 = moderate problems, 4 = severe problems, and 5 = unable/extreme problems). The EQ-5D-5L helps overcome problems with ceiling effects and has greater sensitivity [24]. It showed evidence of good sensitivity in our pilot trial [21] and has been the primary outcome measure in other primary care-based multimorbidity trials [28]. Besides being used as the primary outcome measure in the analysis, the EQ-5D-5L will be also used to estimate QALYs for our economic evaluation.

#### Secondary outcome measures

The following are the secondary outcome measures:

- HRQoL at 3, 6, and 12 months after randomisation using the EQ-5D-5L utility score.
- HRQoL at 3, 6, and 12 months and overall using the PROMIS-29 [29].
- Depression severity at 3, 6, and 12 months and overall using the Patient Health Questionnaire-8 [30].
- Anxiety severity at 3, 6, and 12 months and overall using the Generalised Anxiety Disorder-7 [31].
- Loneliness at 3, 6, and 12 months and overall. Four questions are used to capture different aspects of loneliness. The first three questions are from the University of California, Los Angeles (UCLA) 3-item loneliness scale [32]. The wording of the UCLA questions and response options are taken from the English Longitudinal Study of Ageing. The last is a direct question about how often the respondent feels lonely.
- The incidence of falls over 12 months assessed via self-report at 3, 6, and 12 months.
- Adverse events over 12 months.
- Resource use data will be collected to inform the economic evaluation from patient questionnaires and GP practice records (i.e. medication data).

#### Process evaluation

A qualitative process evaluation will be undertaken to (i) identify, describe, and explain the barriers and facilitators to set-up, recruitment, and trial processes; (ii) describe recipients’ and providers’ experiences of the yoga intervention and study process; and (iii) identify optimal implementation strategies for embedding and normalising the yoga intervention in preparation for a wider roll-out. Data will be collected during the pilot and main trial phases, via semi-structured interviews with both intervention and control group trial participants (*n* = 18–20), trial decliners (*n* = 3–4), and yoga teachers (*n* = 10–12), as well as from observations of standardisation training sessions and yoga classes. A subset of trial participants (*n* = 8–12) will be invited to take part in a second interview approximately 6 months after their first interview, to explore any long-term impact of their trial participation. Pilot phase yoga teachers will be interviewed once per 12-week course they deliver, and their feedback used to optimise the standardisation training and intervention delivery for main phase yoga teacher, main phase teachers will be interviewed once. A subset of trial staff (*n* = 1–5) may also be interviewed to inform intervention implementation. Interviews will be conducted face-to-face or by telephone or video conference.

#### Other outcomes

Adherence to the supervised GYY classes will be recorded by the yoga teachers using class attendance registers. We will also ask participants to report any other supervised or self-managed yoga practice in the follow-up questionnaires. Treatment fidelity will be assessed via observation of a yoga session at each site by the yoga intervention supervisors (LB and JH) and through discussions with teachers as part of the process evaluation. Socio-demographic measures (age, gender, ethnicity, residential status, employment status, smoking status) and details of medical conditions (assessed using an adapted Bayliss measure of illness burden [33]) will be collected at baseline. Participant beliefs and preferences for the GYY programme and usual care will be assessed at baseline and 12 months.

### Participant timeline {13}

**Fig. 2:**
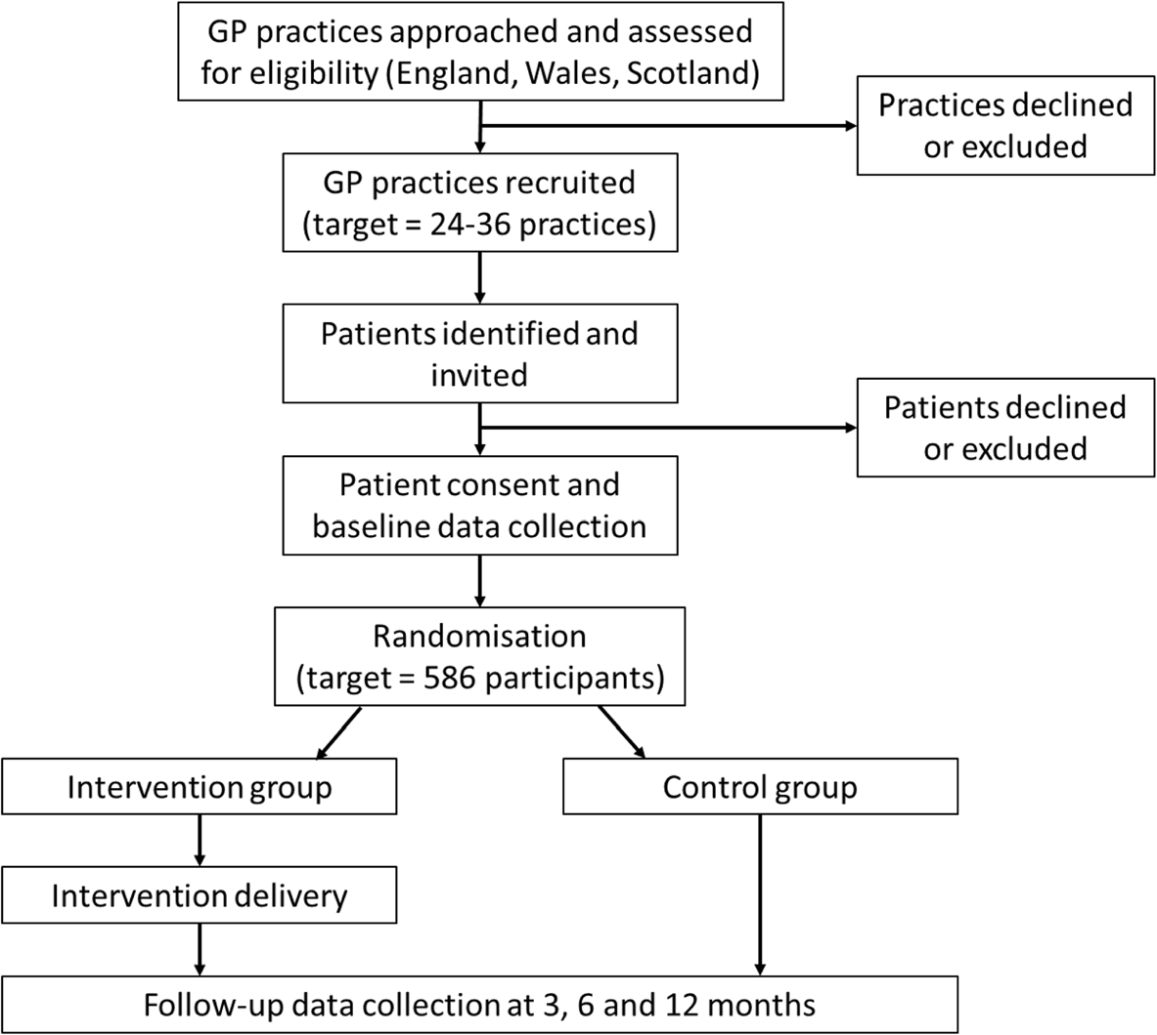
Flow diagram illustrating the practice and patient recruitment, intervention delivery, and follow-up

See Fig. 2 for the participant’s timeline through the trial.

### Sample size {14}

Walters and Brazier [34] in a review paper of the EQ-5D-3L found a difference of 0.074 (mean) or 0.081 (median) to be a minimum clinically important difference among a variety of patients, whilst McClure and colleagues found a difference of 0.063 (mean) or 0.064 (median) for the EQ-5D-5L using simulated data [35]. To be conservative, we took the lowest estimate (0.06) with an estimated standard deviation of 0.20 [21]. Accounting for loss to follow-up of 20%, we need to recruit and randomise 586 participants for the study to have 90% power with 5% significance (two-sided).

Although this is an individually randomised trial, there is a possibility of potential clustering within the intervention arm by yoga class. If we assume an intraclass correlation coefficient of 0.04 and an average class size of 12 in the intervention arm, then with the proposed sample size, we would still retain 84% power to detect the same magnitude of effect (ceteris paribus). In this calculation, we considered the level of clustering at the yoga class level, rather than at the level of the yoga teacher, since we believe this will be the most influential level of clustering. Accounting for potential clustering within the intervention arm only leads to small reductions in power, which could potentially be recovered in the analysis of the repeated measures which is not currently accounted for in the sample size calculation.

### Recruitment {15}

#### Recruitment of practices

General practices that are potentially interested in taking part in the trial will be identified with help from the NHS Clinical Research Networks in England, the Health and Care Research Wales Support and Delivery Centre, and the Scottish Primary Care Network. These nationwide networks facilitate clinical research by identifying and recruiting general practices and providing resources to help practices do research. Trial coordinators will liaise with key stakeholders at the practice (e.g. practice manager, GPs) to explain the requirements of the study. The practice manager or lead GP will sign a practice-level agreement.

#### Recruitment of participants

The main method of participant recruitment will involve inviting potentially eligible patients who are identified from searching general practice databases at approximately 24–36 practices. Other methods that may be used if required include local media advertising, recruiting from patient networks, and inviting potentially eligible patients from previous studies that have been coordinated by York Trials Unit.

For the main recruitment method, each participating practice will be asked to search their practice database using an electronic search process provided by the research team to identify potentially eligible patients who are aged 65 years or older and have two or more chronic conditions as defined by the inclusion criteria. If there are more potentially eligible patients than required (e.g. > 500 for a recruitment mail-out from a single practice required to cover one course/site), a sub-sample of these patients will be randomly selected. GPs will be asked to review the resulting list to screen out patients meeting the exclusion criteria. The practice or Docmail (a third-party information handler) will mail out a recruitment pack to the remaining patients, which will include a covering invitation letter, a participant information sheet (which includes a link to an audio version on the trial website), a consent form, a screening questionnaire, and two prepaid envelopes for returning the completed forms. If the patient is screened as eligible, a study investigator will notify the patient’s GP and ask them to confirm the patient’s suitability for participation. Eligible patients will then be sent a baseline questionnaire to complete and return by a specified date. This questionnaire will collect data on socio-demographics measures, primary and secondary outcome measures, and preferences/beliefs for the treatments on offer in the trial.

##### Alternative process due to COVID-19 restrictions

The COVID-19 pandemic has made the above postal consent and questionnaire data collection processes difficult to implement because the trial team has been required to work from home. Therefore, where necessary, consent will be completed electronically by potential participants via an online form, participants will be contacted by a study investigator to collect screening and baseline questionnaire data via telephone, and this questionnaire data will be entered electronically via an online form by a study investigator.

#### Recruitment of yoga teachers

Yoga teachers who are potentially interested in taking part in the trial will be identified by the trial’s yoga consultants (LB and JH). The consultants are aware of who has completed the GYY teacher training course and where they are based. The consultants and trial coordinators will liaise with potential teachers to explain the requirements of the study. Each teacher will sign a contract with the University of York.

### Assignment of interventions: allocation

#### Sequence generation {16a}

The randomisation is stratified by site using varying block sizes and allocation ratios.

#### Concealment mechanism {16b}

Participants will be randomised via a central, computer-based randomisation system, designed and managed by York Trials Unit. Since a group of participants is assigned to a treatment group in one go as opposed to being randomised one-by-one, as described in the next section, the allocation sequence cannot be predicted in advance.

#### Implementation {16c}

When enough patients (ideally 20–30) have provided baseline data and stated their availability for a particular GYY course at a particular site, they will be randomised collectively by a member of the research team using the randomisation system. The block of patients will be allocated to either the intervention or control group in a ratio that can vary and is specified to ensure that the maximum class size is not exceeded (12–15 participants for face-to-face and 12 for online to the intervention group, and the rest to control). We are targeting an overall allocation ratio of 1:1.

### Assignment of interventions: blinding

#### Who will be blinded {17a}

Due to the nature of the intervention, participants and yoga teachers will not be blinded to the group allocation. Outcome measures are primarily self-reported. Members of the research team collecting the outcome data over the telephone will take reasonable steps to ensure that they are blind to the participant’s group allocation.

Although GPs will be informed about study participation, they will not be informed about allocation status, reducing the risk of inducing GP behaviour change based on this knowledge. The wider health and social care team will not be informed about study participation or allocation status.

#### Procedure for unblinding if needed {17b}

This is primarily an open trial, so there are no specific emergency unblinding procedures. Group allocation may, if necessary, be revealed to a participant’s GP in response to a yoga-related adverse event.

### Data collection and management

#### Plans for assessment and collection of outcomes {18a}

Data will be collected via participant-completed questionnaires at baseline and at 3, 6, and 12 months after randomisation. Completed outcome data are returned primarily by post but also via telephone where necessary (e.g. for participants who require assistance to complete their questionnaires). Demographic and medical history information will be collected at baseline. All participants will be given a diary to prospectively record their health service use to aid them with completing the health service use section in follow-up questionnaires.

#### Plans to promote participant retention and complete follow-up {18b}

The following plans will be implemented to promote participant retention and complete follow-up:

- Yoga teachers will encourage regular class attendance and contact participants who miss two consecutive classes without prior notification.
- Participants who withdraw from the yoga programme may choose to remain in the trial to provide follow-up data.
- Participants will receive a £5 shopping voucher or £5 cash with every follow-up questionnaire as a thank you for their continuing participation in the trial.
- A text message will be sent to participants who have consented to receive this form of communication 1–7 days before each questionnaire is sent, saying that they will shortly receive a questionnaire and to complete and return it as soon as possible. The text message also acts as a prompt for the participant to inform the research team if they have moved address.
- A reminder letter will be sent if the questionnaire has not been returned within 14–21 days.
- A researcher will call the participant to complete the questionnaire over the phone if it has not been returned within 28 days.
- If a questionnaire is returned incomplete or with errors, a researcher will call the participant for clarification or completion of missing or invalid data.
- Participants will be advised that they are able to phone a member of the research team if they require assistance with completing a questionnaire.
- A newsletter containing information about trial progress and any relevant updates will be sent out to participants via post or email every 3–6 months.
- After completing the final follow-up questionnaire (12 months), all control participants will receive details of GYY or other suitable yoga classes that they could join on a self-pay basis.
- After randomisation to the main trial, participants allocated to the control group will be randomised again to receive the offer of a one-off group yoga class, which will take place when the final follow-up is completed, or no offer.

#### Data management {19}

Case report forms will be used to record all the information required from the protocol. Essential trial documentation which individually and collectively permits evaluation of the conduct of the trial and the quality of the data produced will be kept within the Trial Master File. The sponsor will ensure that this documentation is retained for a minimum of 5 years after the conclusion of the trial and a minimum of 20 years in electronic format in accordance with the guidelines on Good Research Practice. Paper data will then be disposed of securely and electronic data will be anonymous of identifiable information.

All study-related information will be stored securely in the coordinating centre at the University of York or at an alternative secure off-site facility. All electronic records will be stored on a password-protected server. All participant data will be identified by a coded identification number to maintain participant confidentiality.

All participant information will be stored in locked cabinets in areas with restricted access. Process evaluation data (e.g. interview audio recordings) will be stored securely on a password-protected server at Northumbria University. Participant data may only be reviewed by authorised persons on the research team or other authorised people to verify that the study is being carried out correctly; all of whom will have a duty of confidentiality. Trial participants will give permission for this authorised review of their data at the time of consent. All names and other identifying information will be removed before the data is analysed and the results presented at conferences and in scientific journals.

#### Confidentiality {27}

Data will be handled in accordance with the Data Protection Act 2018, GDPR legislation, the latest Directive on Good Clinical Practice, and local policy.

#### Plans for collection, laboratory evaluation, and storage of biological specimens for genetic or molecular analysis in this trial/future use {33}

No biological specimens will be collected.

### Statistical methods

#### Statistical methods for primary and secondary outcomes {20a}

Statistical analyses will be described in detail in a statistical analysis plan (SAP) that will be agreed upon with the Trial Steering Committee before all data has been collected. Any subsequent amendments will be clearly stated and justified. The trial will be reported in accordance with the Consolidated Standards of Reporting Trials (CONSORT) guidelines for clinical trials [36]. Baseline data will be summarised using descriptive statistics with no formal statistical comparisons of the baseline data undertaken. All analyses will be conducted on an intention-to-treat basis and using two-sided significance tests at the 5% significance level, unless otherwise stated.

The primary outcome will be analysed using a linear mixed model, including all available follow-up time points. The model will adjust for EQ-5D-5L at baseline and include as fixed effects: time point, trial arm, arm by time interaction, and other covariates specified in the SAP. Patient (to account for the repeated measures) and site will be included as random effects. The overall difference between the two groups over the 12 months from randomisation will be the primary endpoint, but differences at each time point will be extracted for secondary investigations aimed at determining the potential pattern of change. Adjusted mean differences will be presented with an associated 95% CI and *p* value. Different covariance patterns for the repeated measurements will be explored, and the most appropriate pattern will be used for the final model. Model assumptions will be checked, and if they are in doubt, transformations of the outcome data will be considered. Analyses to account for possible clustering by yoga class will also be undertaken by including the class as a random effect, nested within the treatment arm. We shall explore the impact of differing modes of delivery (face-to-face, or online) within subgroup analyses.

The anxiety, depression, loneliness, and PROMIS-29 data will be analysed in the same way as described for the primary outcome. The number of falls experienced by participants over the 12 months will be analysed using a negative binomial regression model. The number and type of serious and non-serious adverse events will be summarised descriptively by randomised group.

#### Interim analyses {21b}

There are no planned interim analyses for the trial or stopping guidelines. There is, however, an internal pilot study from which data will contribute to the final analyses. The primary reason for this pilot study will be to check the assumptions about recruitment and feasibility of the trial. The period covering the two waves of yoga courses at the four pilot sites will form the internal pilot phase. Descriptive statistics will be used to evaluate the four following progression criteria and inform study continuation beyond the pilot phase:

1. Intervention provision
  - Green: 3–4 sites offering their first group yoga session within 3 weeks after randomisation
  - Amber: 1–2 sites offering their first group yoga session within 3 weeks after randomisation
  - Red: 0 sites offering their first group yoga session within 3 weeks after randomisation
2. Intervention acceptability
  - Green: ≥80% for overall class attendance rate
  - Amber: 65–80% for overall class attendance rate
  - Red: < 65% for overall class attendance rate
3. Recruitment
  - Green: 3–4 sites recruited ≥20 patients each within 4 months
  - Amber: 1–2 sites recruited ≥20 patients each within 4 months
  - Red: 0 sites recruited ≥20 patients each within 4 months
4. Six-month follow-up
  - Green: ≥80% completion of the EQ-5D-5L
  - Amber: 65–80% completion of the EQ-5D-5L
  - Red: < 65% completion of the EQ-5D-5L

If any criteria are graded as amber, a rescue plan will be developed outlining steps to be taken to improve intervention provision, recruitment, class attendance, and/or outcome follow-up (as appropriate) and will be agreed upon with the Trial Steering Committee and the funder. If all the progression criteria are failed (red), then the trial will not continue beyond the pilot phase.

### Methods for additional analyses (e.g. subgroup analyses) {20b}

#### Process evaluation analysis

Data analysis will be ongoing and iterative throughout the trial. Interviews will, with consent, be audio-recorded, transcribed verbatim, and edited to ensure respondent anonymity. Contemporaneous field notes from observations will also be edited to ensure participant anonymity. The analysis will be theoretically informed by the Normalisation Process Theory [37, 38] and will be conducted according to the standard procedures of rigorous qualitative analysis [39] including open and focused coding, constant comparison, memoing [40], deviant case analysis [41], and mapping [42]. Independent coding and cross-checking will be conducted, and a proportion of data will be analysed collectively in ‘data clinics’ where the process evaluation team share and exchange interpretations of key issues emerging from the data.

The process evaluation team will present anonymised emerging findings to the Trial Management Group on the potential determinants of trial set-up and recruitment. These might include site-specific issues, issues across multiple sites, or at the level of the organisation of the trial. Where necessary, the process evaluation team will work with the Trial Management Group and specific sites to develop and implement action plans. The focus will be on aspects of trial management and delivery that are amenable to change and all feedback will be supportive and constructive.

#### Cost-effectiveness analysis

The economic analysis will assess the relative cost-effectiveness of the GYY programme in addition to continued access to usual care compared with usual care alone. Costs and health outcomes will be evaluated from the perspective of the NHS and Personal Social Services, consistent with that used by NICE [43]. Data on healthcare resource use, intervention costs, and health outcomes will be collected during the 1-year trial period. Costs components will comprise primary and social care consultations (e.g. with a GP, nurse, or physiotherapist), hospital visits (e.g. inpatient episodes, outpatient visits, and accident and emergency admissions), and private treatments. Prescription data will also be obtained from GP practice records for up to 100 participants and covering the 15-month period before the 12-month follow-up. Trial records will be used to estimate the cost of the yoga intervention. Private expenditures related to treatment will also be recorded, and these costs will be included in a secondary analysis. We will estimate the cost per participant by multiplying the use of resource use by their associated unit cost, which will be valued using national published sources such as NHS reference costs and the British National Formulary. Health outcomes will be expressed in terms of the QALY using the EQ-5D data collected at baseline and 3, 6, and 12 months of follow-up. QALYs will be calculated by plotting the utility scores at each follow-up point and estimating the area under the curve [44]. Costs and QALY data will be synthesised to generate an incremental cost-effectiveness ratio (ICER), which is defined as the ratio of the mean difference in costs to the mean difference in QALYs between both arms of the trial. For the analysis, regression methods will be used to allow for differences in prognostic variables. The pattern of missing data will be analysed and handled by means of multiple imputations if deemed appropriate according to the missing data pattern in the dataset [45, 46]. Sensitivity analyses will be conducted to test the robustness of the results, including probabilistic sensitivity analysis. The uncertainty will be presented using a cost-effectiveness acceptability curve, which shows the probability of the yoga intervention plus usual care being more cost-effective than usual care alone. The probability that each intervention is cost-effective will be reported at the cost-effectiveness thresholds applied by NICE of £20,000 to £30,000/QALY [43] and also £13,000/QALY as suggested by recent research [47, 48]. A cost-consequence analysis will also be conducted to capture the wider consequences of the yoga intervention in terms of the full breadth of outcomes measured in the trial. A detailed health economics analysis plan will be agreed upon with the Trial Steering Committee before all data has been collected.

### Methods in analysis to handle protocol non-adherence and any statistical methods to handle missing data {20c}

In the primary clinical analysis, the impact of missing EQ-5D-5L outcome data will be minimised to some extent by using the mixed-effects, repeated measures model, as it allows the inclusion of intermittent responders under the assumption that data are missing at random. Nevertheless, the extent and pattern of missing data for each outcome will be explored and predictors of missingness examined, especially if these vary by intervention. If necessary, multiple imputations will be utilised to impute missing data and explore deviations from the missing at random assumption.

Complier average causal effect (CACE) analyses for the primary outcome will be undertaken to explore the impact of non-compliance on treatment effect estimates. Three analyses are proposed. The first CACE analysis will be conducted on the data of participants who are fully compliant, defined as attendance at three or more of the first six sessions and at least three other sessions. The second CACE analysis will define compliance as attendance at one yoga session or more (i.e. any compliance), which will include participants who are fully and partially compliant. The final CACE analysis will include the number of sessions attended in its continuous form.

### Plans to give access to the full protocol, participant level-data and statistical code {31c}

The full protocol is available via the funder website: https://fundingawards.nihr.ac.uk/award/17/94/36. Requests for other data or documentation should be made by contacting the corresponding author.

### Oversight and monitoring

#### Composition of the coordinating centre and trial steering committee {5d}

The trial coordination team meets regularly with the chief investigator, yoga consultants, and process evaluation team. A Trial Management Group and Trial Steering Committee will oversee the project. The Trial Management Group will typically meet every 2 months and comprises the chief investigator, the co-applicant co-investigators, the trial and data coordinators, a process evaluation researcher, a clinician, a patient representative, and a BWY representative. The Trial Steering Committee will meet approximately every 6–9 months and includes an independent chairperson and members as well as representatives of the Trial Management Group (chief investigator, statisticians, trial coordinators).

#### Composition of the data monitoring committee, its role and reporting structure {21a}

The Trial Steering Committee has agreed to additionally take on the role of the data monitoring committee. A separate data monitoring committee was considered unnecessary due to the low-risk and open nature of the trial. The Trial Steering Committee will review and discuss data pertaining to participant safety and trial progress.

#### Adverse event reporting and harms {22}

Adverse events related to yoga practice or study participation during the 12 months after randomisation will be recorded by trial coordinators following assessment of seriousness, causality, and expectedness by the chief investigator and a clinician co-investigator. We will also record all deaths and all falls that result in hospitalisation, regardless of causality. Yoga teachers and participants are responsible for notifying the trial office of any adverse events. The chief investigator or delegate will report any related and unexpected serious adverse events to the Research Ethics Committee within 15 days of the chief investigator becoming aware of it. Adverse events will be summarised and reported to the Research Ethics Committee, the funder, and the Trial Steering Committee in their regular progress reports.

#### Frequency and plans for auditing trial conduct {23}

The trial office and sponsor monitor the aspects of the study on an ongoing basis. The Trial Steering Committee will meet approximately every 6–9 months.

#### Plans for communicating important protocol amendments to relevant parties (e.g. trial participants, ethical committees) {25}

Important protocol modifications are those that are likely to significantly affect the safety of the participants, the scientific value of the study, or the conduct or management of the study. These substantial amendments will be submitted to the Research Ethics Committee for approval having been agreed with the funder, sponsor, Trial Steering Committee, and Trial Management Group. Minor modifications to the protocol will be agreed upon with the Trial Management Group and sponsor before submission for approval to the Research Ethics Committee. All amendments will be implemented following the guidance of the Health Research Authority. Trial participants will be written to, where necessary, to explain any changes. All amendments whether substantial or not will be listed in the published final report to the funder.

#### Dissemination plans {31a}

We will develop a publication and dissemination plan to include conference presentation(s) and journal publication(s). We will send a written summary of the trial results to all participants who request this on their final follow-up questionnaire. We will also plan dissemination to the relevant patient and clinical interest groups.

This protocol is being made publicly available. It is planned for a full trial report to be submitted to the funder and for publication in a peer-reviewed journal. The full trial report will be open access and made available as a permanent archive in the NIHR Journals Library. At the time of publishing the protocol, there was no plan to make the anonymised participant-level dataset and statistical code for generating the results publicly available. However, external requests for this data and code will be considered by the Trial Management Group after the publication of the main trial findings.

The criteria for authorship and contributorship will be taken from the International Committee of Medical Journal Editors [49]. Those who did not design the study or contribute to drafting the work but were involved in the trial conduct (e.g. yoga teachers and staff at recruiting practices) will be acknowledged as collaborators. When a journal permits, we will list all authors rather than use a group name. There will be a designated writing group for each publication and one or more lead writers who convene the group. Any member of the trial team can propose a publication to the chief investigator, lead trial coordinator, and senior member of the York Trials Unit. For each publication, all members of the trial team will be asked whether they consider themselves to be a potential author, contributor, or neither. The chief investigator, lead trial coordinator, and senior member of the York Trials Unit will then agree on the attributions. There are no plans to use professional medical writers to assist with the preparation of trial reports or publications.

## Discussion

Multimorbidity is common in older adults and associated with high levels of illness burden and healthcare expenditure [4–7]. We know from previous work and from reviews that there is a lack of evidence-based interventions for older adults with multimorbidity [8, 9]. Yoga might be a useful intervention because it is low-cost, simple, and can address several medical conditions simultaneously. However, many of the previous research studies on yoga have limitations, including small sample sizes, a single yoga teacher delivering the programme, and short-term follow-up. In addition, few studies have focused on older adults with multimorbidity. We have successfully completed a pilot trial that demonstrated the feasibility and acceptability of the GYY programme in this population [21]. The current full-scale trial will build on this previous work by evaluating the clinical and cost-effectiveness of this intervention.

Should the trial demonstrate the GYY programme to be clinically and cost-effective, we will have the necessary information from our process evaluation to identify optimal implementation strategies for embedding and normalising the intervention beyond the study. Easy integration into routine care pathways should be possible, potentially using models and referral processes adapted from those used in exercise referral and social prescribing schemes.

### Trial status

Recruitment to the trial opened on 16 August 2019 and is due to end in September 2021 with follow-up completed by the end of September 2022. The current protocol is version 1.5 (dated 09/07/2020).

## Abbreviations

BWY: British Wheel of Yoga
BWYQ: British Wheel of Yoga Qualifications
CACE: Complier average causal effect
CI: Confidence interval
CONSORT: Consolidated Standards of Reporting Trials
EQ-5D: EuroQol 5 Dimensions
GDPR: General Data Protection Regulation
GP: General practitioner
GYY: Gentle Years Yoga
HRQoL: Health-related quality of life
ICER: Incremental cost-effectiveness ratio
ISRCTN: International Standard Randomised Controlled Trial Number
MD: Mean difference
NICE: National Institute for Health and Care Excellence
NHS: National Health Service
NIHR: National Institute for Health Research
PROMIS-29: 29-item Patient-Reported Outcomes Measurement Information System®
QALY: Quality-adjusted life year
SAP: Statistical analysis plan
SMD: Standardised mean difference
UCLA: University of California, Los Angeles
